# Morphology and Glycan Composition of the Mandibular Glands in the White‐Eared Opossum (*Didelphis albiventris*)

**DOI:** 10.1002/jmor.70074

**Published:** 2025-08-16

**Authors:** Bruno Cesar Schimming, Aline Herrera Farha, Tais Harumi de Castro Sasahara, Fabio Cesar Magioli Abdala, Attilio Cianciotta, Silvio Pires Gomes, Salvatore Desantis

**Affiliations:** ^1^ School of Veterinary Medicine and Animal Science, Graduate Program in Wild Animals, Botucatu São Paulo State University (UNESP) São Paulo Brazil; ^2^ Institute of Biosciences of Botucatu, Laboratory of Wildlife Anatomy Botucatu, São Paulo State University (UNESP) São Paulo Brazil; ^3^ School of Veterinary Medicine and Animal Science, Graduate Program in Anatomy of the Domestic and Wild Animals São Paulo University (USP) São Paulo Brazil; ^4^ Veterinary Clinics and Animal Production Unit, Department of Precision and Regenerative Medicine and Ionian Area University of Bari Aldo Moro Bari Italy

**Keywords:** glycans, lectins, marsupials, salivary glands

## Abstract

The white‐eared opossum, *Didelphis albiventris*, is an opportunistic and omnivorous marsupial, whose diet ranges from wild fruits to eggs and birds. Salivary glycoproteins play a key role in the protection of the oral cavity and the formation of the food bolus. Despite the importance of salivary glycoproteins, their detailed investigation in the white‐eared is lacking. This study investigated the morphology and glycan composition of the mandibular salivary glands of the white‐eared opossum for the first time. Histological and histochemical investigations were conducted on tissue fragments fixed with 4% PBS‐buffered paraformaldehyde and embedded in Paraplast. The pattern of glycoproteins was investigated using traditional histochemical methods (PAS, Alcian Blue pH 2.5, and High‐Iron Diamine staining) and lectin histochemistry. The glandular parenchyma consisted of acinar secretory units and a duct system characterized by abundant striated ducts. Secretory acini secrete neutral glycans and non‐sulfated acid glycans. Mannosylated N‐linked glycans terminating in α2,6‐sialic acid and fucose are expressed in the secretory acini, containing intraluminal α2,3‐sialylated O‐linked glycans. The epithelial lining of the striated and interlobular ducts also shows O‐linked glycans with terminal Galβ1, 3GalNAc, and *α*GalNAc residues. Finally, the epithelium and lumen of interlobular ducts are enriched with additional GalNAc‐terminated O‐linked glycans with the appearance of lactosaminated glycans and the disappearance of α2,3‐sialylated glycans. These results suggest that the saliva produced by the mandibular gland of the white‐eared opossum consists of a species‐specific pattern of glycoproteins, to whose composition the ductal system also contributes. The observed glycan composition is probably related to the diet of the white‐eared opossum and its adaptations to the environment and food availability. These results indicate that the mandibular salivary gland of the white‐eared opossum *Didelphis albiventris* has specific histological and molecular characteristics compared to other marsupial species, suggesting that diet and habitat, but not the taxonomic group, influence the mandibular gland features.

## Graphical abstract

This study analyzed the morphology and glycan composition of the mandibular salivary glands of the white‐eared opossum (*Didelphis albiventris*) via histological and glycohistochemical investigations. The glandular parenchyma consisted of acinar units and a duct system with abundant striated ducts. Secretory acini reacted with PAS, Alcian Blue, Con A, MAL II, and SNA positive glycans. The ducts showed additional glycan reactivity for several lectins including RCA and SNA. A species‐specific glycoprotein pattern and the contribution of the ducts is hypothesized.

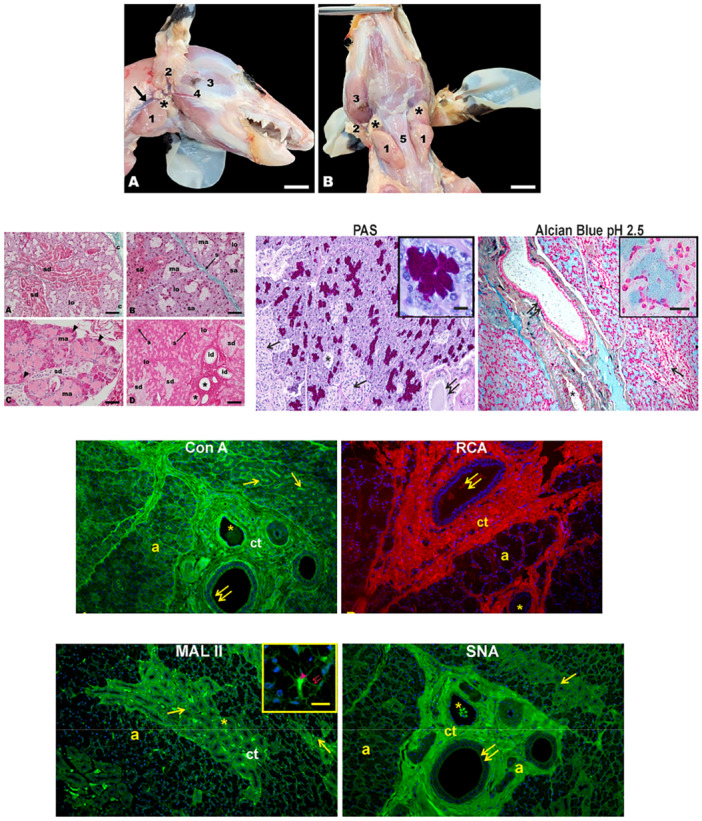

## 1 Introduction

The salivary glands belong to the digestive system, and their ducts release the saliva into the oral cavity. The saliva moistens and maintains the health of the oral cavity and supports physiological functions such as chewing, lubrication, swallowing, deglutition, and the initiation of food digestion in the oral cavity (Sisto et al. 2019; Kumar et al. 2017; Hamphery and Williamson 2021).

The salivary glands are categorized as minor and major salivary glands. The minor salivary glands are located in the lamina propria of the oral mucosa, whereas the major salivary glands are extramural glands.

The major salivary glands in mammals include the mandibular, parotid, and sublingual glands (Massoud et al. 2023). Other animals also may have zygomatic glands such as bushy‐tailed opossums (Vieira et al. 2015), dogs (Gil et al. 2018), agoutis (Oliveira Júnior et al. 2016) and spix's yellow‐toothed cavy (Rebouças et al. 2024).

In general, the morphology of salivary glands is species‐specific and depends on the combined influence of various factors such as diet, habitat, and taxonomic affiliation. For example, the mandibular gland, which is the focus of the present study, is rounded in the crab‐eating raccoon (*Procyon cancrivorus*) (Pereira et al. 2013), in the crab‐eating fox (*Cerdocyon thous*) (Pereira and Faria Júnior 2018), and the capybara (*Hydrochoerus hydrochaeris*) (Donoso et al. 2024) but has an oval shape in koala (*Phascolarctos cinereus*) (Krause 2010), in the Eurasian wolf (*Canis lupus lupus*) (Klećkowska‐Nawrot et al. 2025), in the Echidna (*Tachyglossus aculeatus*) (Monotremata) (van Lennep et al. 1979), in water buffaloes (*Bubalus bubalis*) (Nourinezhad et al. 2021) and the bushy‐tailed opossum (*Glironia venusta*) (Vieira et al. 2015). An elliptical‐pyramidal mandibular gland is found in the southernwhite‐breasted hedgehog (*Erinaceus concolor*) (Massoud et al. 2023) while it is pyramidal in the giant anteater (*Myrmecophaga tridactyla*) (Schimming et al. 2025). Finally, the porcupine (*Hystrix indica*) (Goodarzi and Kahrizi 2023), and spix's yellow‐toothed cavys (*Galea spixii* Wagler, 1831) (Rebouças et al. 2024) show almost rectangular and triangular mandibular glands, respectively.

Saliva is a complex aqueous fluid that contains electrolytes, enzymes, and glycoconjugates such as glycoproteins, which constitute a dense mucous film that contributes significantly to the viscoelastic properties of saliva and oral defense by interacting with oral microorganisms and modulating the beneficial microbiota and eliminating the pathogenic microbiota (Carpenter 2013; Cross and Ruhl 2018). In addition, salivary glycans are involved in food bolus formation (Gao et al. 2022; Kozak et al. 2016).

The in situ detection of glycans is carried out using histochemistry (PAS, Alcian blue, HID) and lectin histochemistry. These methods reveal the presence of neutral and acidic mucins. Lectin histochemistry explores the glycan profile of cells and tissues because lectins distinguish specific sugars in structurally different glycans (Brooks 2024; Spicer and Schulte 1992).

Histochemistry has been used to study the mandibular gland of (i) carnivores such as ferret (*Mustela furo*) (Poddar and Jacob (1977) and Eurasian wolf (*Canis lupus lupus*) (Klećkowska‐Nawrot et al. 2025); (ii) herbivores such as porcupine (*Hystrix indica*) (Goodarzi and Kahriz 2023), Koalas (*Phascolarctos cinereus*) (Krause (2010), southern white‐breasted hedgehog (*Erinaceus concolor*) (Massoud et al. 2023); (iii) myrmecophagic animals such as armadillo *Zaedyus pichiy* (Estecondo et al. 2005), lowland tapir (*Tapirus terrestris Perissodactyla*) (Klećkowska‐Nawrot et al. 2023), aardvark (*Orycteropus afer* Tubulidentata) (Klećkowska‐Nawrot et al. 2023), giant anteater (*Myrmecophaga tridactyla* Linnaeus, 1758) (Schimming et al. 2025), nine‐banded armadillo (*Dasypus novemcinctus*) (Shackleford 1963), echidna (*Tachyglossus aculeatus*) (van Lennep et al. 1979), and (iv) omnivores such as Australian possum (*Trichosurus vulpecula*) (Blood et al.^1977^), opossum (*Didelphis virginiana*) (Pinkstaff 1975), and pig (*Sus domesticus*) (Pedini et al. 2000).

It has been reported that salivary glycoproteins belong to N‐ and O‐linked glycans that can be sulfated, fucosylated, and sialylated (Donohue et al. 2011; Gao et al. 2022; Kozak et al. 2016). Terminal sialic acids are particularly important for saliva function because their negative charges impart viscoelastic properties to saliva and regulate interactions between salivary mucins and the oral microbiota (Cross and Ruhl 2018). Therefore, the use of lectin histochemistry is a good tool to map the glycosylation of the mandibular gland and its secreted material. This methodology has been used to investigate in situ the glycoprofile in the mandibular glands of the dog (Pedini et al. 1994), fallow‐deer (Pedini et al. 1997), pig (Pedini et al. 2000), horse (Scocco and Pedini 2006), and giant anteater (Meyer et al. 1993; Schimming et al. 2025). Lectins have been used to study the composition of a mucous glycoprotein obtained from the mandibular gland extracts of the armadillo (Wu et al. 1995).

The white‐eared opossum (*Didelphis albiventris*) is widely distributed in Brazil, Paraguay, Uruguay, Argentina, Bolivia, Ecuador, Peru, and Colombia (Sousa et al. 2012). This species is opportunistic and omnivorous. Its diet includes fruits, seeds, leaves, insects, molluscs, and vertebrates of different sizes, although it frequently attacks chicken coops and feeds on eggs and chicks (Flórez‐Oliveros and Vivas‐Serna 2020). The dietary variation of this marsupial characterizes the importance of the species in the food chain, in addition to contributing to adaptation to different environments (Jorge et al. 2012; Nascimento et al. 2019). The white‐eared opossum is frequently observed in rural and urban environments. Several reports suggest that the dietary flexibility of white‐eared opossums contributes to their wide geographic distribution and survival in environments impacted by human activity (Albert et al. 2020; Bitencourt and Bezerra 2022; Delciellos et al. 2016).

The ecological importance of the species is emphasized by its ability to coexist in urban environments and contribute to dispersing viable seeds via defecation, playing a crucial role as dispersers in urban forest fragments (Cáceres 2002; Cáceres and Monteiro‐Filho 2007; Oliveira and Leme 2013). Due to the ecological importance related to the dietary adaptation of the white‐eared opossum, our research group is studying the morphological, histological and glycohistochemical characteristics of its major salivary glands (Farha et al. 2025).

To the best of the authors' knowledge, there are no reports on the morphology and the glycan composition of the mandibular gland in the white‐eared opossum (*Didelphis albiventris*). Thus, the objective of this study was to examine the histological features and the glycan pattern of the mandibular gland of the white‐eared opossum and compare the results with those of other marsupial and eutherian mammals.

## 2 Materials and Methods

### 2.1 Animals

Tissue fragments of mandibular glands from eight (five males and three females) adult white‐eared opossums, *Didelphis albiventris* Lund, 1840 were used. The marsupials were donated by the Centre for Medicine and Research in Wild Animals (CEMPAS), School of Veterinary Medicine and Animal Science, UNESP, Botucatu, São Paulo, Brazil. The opossums were donated after death, for causes unrelated to this study. The investigation was carried out on animals received during the spring and summer seasons, when they are most active. No impact of season or sex was observed in the results of this study. This study was authorized by the Committee on the Use of Animals of the Biosciences Institute of Botucatu, São Paulo State University, UNESP, Botucatu, São Paulo, Brazil (CEUA n° 7380270922/2022).

### 2.2 Anatomical and Light Microscopical Studies

The marsupials were divided into two groups: group A (*n* = 2) and group B (*n* = 6). The animals in group A were used for anatomical studies. The animals were taken to the Laboratory of Wildlife Anatomy, Institute of Biosciences of Botucatu, UNESP, where they were fixed with 10% aqueous formaldehyde solution injected via common carotid artery and immersed in the fixative solution for a week at room temperature (RT). Afterwards, the mandibular gland was dissected and isolated from adjacent structures and photographed in situ.

The animals in group B were used for light microscopy studies. These animals were dissected, and the mandibular glands were accessed and extracted. Fragments of the mandibular gland were obtained and fixed in 4% paraformaldehyde in 0.1 mol L^−^^1^ Phosphate buffer for 24 h at RT and subjected to routine inclusion in Paraplast (Sigma, St. Louis, MO, USA). Serial sections (5 μM thick) were cut and, after de‐waxing with xylene and hydration in an ethanol series of descending concentrations, were stained with hematoxylin‐eosin (H&E) and Masson's Trichrome for morphological analysis and with histochemical procedures for glycan characterization.

### 2.3 Glycohistochemistry

#### 2.3.1 Conventional Histochemistry

Sections were treated with (1) periodic acid‐Schiff (PAS) reaction for neutral glycans (Mc Manus 1948); (2) Alcian Blue pH 2.5 (AB 2.5) for sulfate esters and carboxyl groups in mucins (Pearse 1968); and (3) combined high iron diamine (HID)/Alcian Blue pH 2.5 (HID/AB 2.5) for simultaneous staining of sulphated and non‐sulphated acidic glycans (Spicer 1965).

#### 2.3.2 Lectin Histochemistry

To characterize the glycan pattern, tissue sections were treated according to Desantis et al. (2022). Briefly, after rinsing in 0.05 mol l^−1^ phosphate‐buffered saline (PBS), pH 7.4, the sections were incubated at RT for 2 h in the dark with appropriate dilutions of eight fluorescent lectins (Table 1) diluted in the PBS. All lectins were obtained from Vector Laboratories (Burlingame, CA, USA). After three rinses in PBS, slides were mounted in VECTASHIELD Antifade Mounting Medium with DAPI (Vector Lab., Burlingame, CA, USA). Each experiment was repeated twice for each sample. Controls for lectin staining included (1) substitution of the substrate medium with buffer without lectin and (2) incubation with each lectin in the presence of its hapten sugar. All control experiments gave negative results.

**Table 1:** Lectin used, their sugar specificities, and the inhibitory sugars used in control experiments.

Lectin abbreviation	Source of lectin	Concentration (µg/mL)	Sugar specificity	Inhibitory sugar
AAL	*Aleuria aurantia*	30	Fucα1,2/α1,3/α1,4/α1,6	Fucose
Con A	*Canavalia ensiformis*	25	αMan	Mannose
DBA	*Dolichos biflorus*	30	αGalNAc	GalNAc
MAL II	*Maackia amurensis*	30	Neu5Acα2,3Galβ1,3GalNAc	NeuNAc
PNA*	*Arachis hypogaea*	30	Galβl,3GalNAc	Galactose
RCA_120_*	*Ricinus communis*	25	Galβ1,4GlcNAc	Galactose
SBA	*Glycine max*	30	α/βGalNAc	GalNAc
SNA	*Sambucu snigra*	30	Neu5Acα2,6Galβ1,3GlcNAc	NeuNAc

*Note:* *Rhodamine‐labeled lectin. Nonmarked lectins are fluorescein isothiocyanate‐labeled lectins. The sugar specifity comes from Bojar et al. (2022).

Abbreviations: Fuc, fucose; Gal, galactose; GalNAc, N‐acetylgalactosamine; Glc, glucose; GlcNAc, N‐acetylglucosamine; Man, mannose; NeuNAc, N‐acetylneuraminic (sialic) acid.

The evaluation of staining intensities was based on subjective estimation of the two authors (S.D., B.C.S.). A high degree of consistency was found among observers. The histochemical evaluation was indicated as follows: ‐, negative reaction; +, weak reaction; ++, moderate reaction; +++, strong reaction. Microphotographs were taken with a digital camera (DS‐U3, Nikon, Japan) connected to a light microscope Eclipse Ni‐U (Nikon, Japan). The images were analyzed by the image‐analysis program NIS Elements BR (Vers. 4.20) (Nikon, Japan), Department of Regenerative and Precision Medicine and Ionica Area, University of Bari Aldo Moro, Valenzano (BA), Italy.

## 3 Results

### 3.1 Morphology of the Mandibular Gland

**Figure 1:**
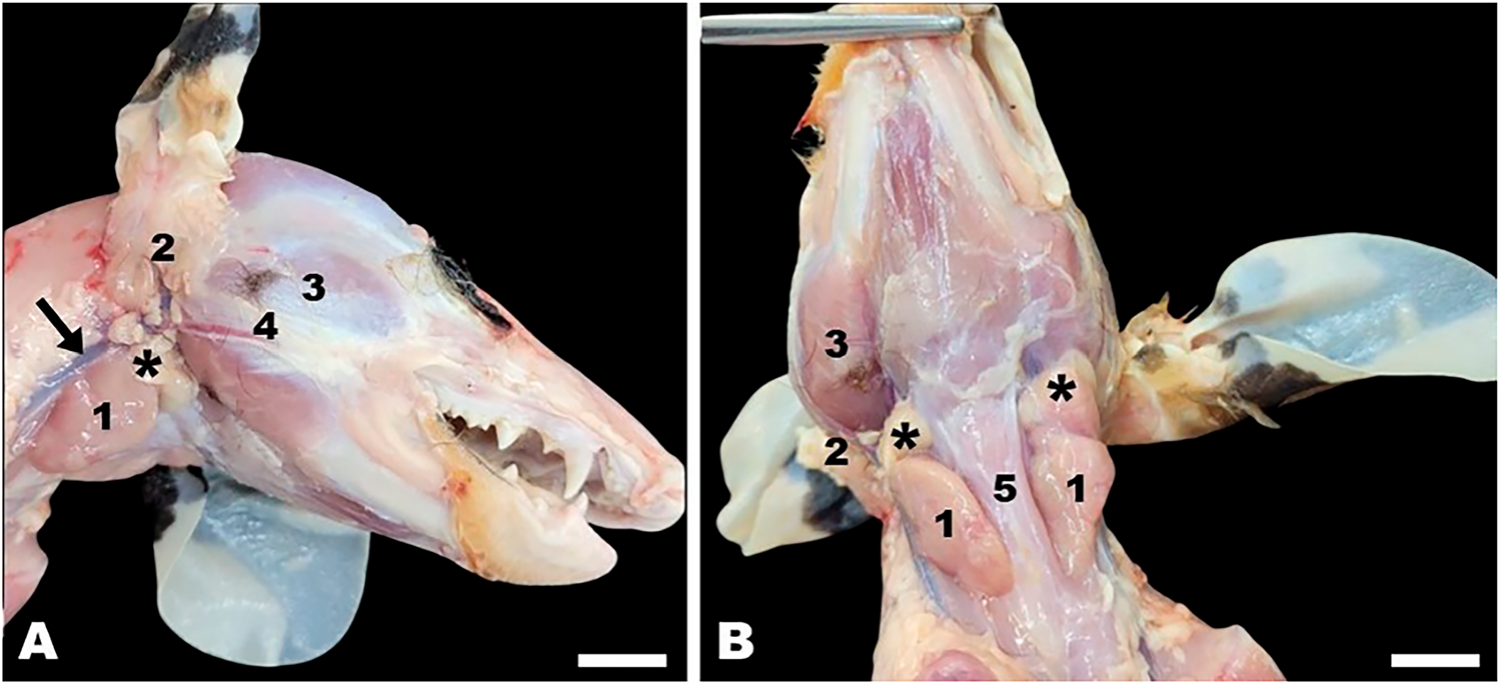
*Didelphis albiventris*, lateral (A) and ventral (B) view of the neck and head regions. Note the mandibular (1) and parotid (2) salivary glands, masseter muscle (3), zygomaticus muscle (4), sternohyoid muscle (5), mandibular lymph nodes (asterisks) and external jugular vein (arrow). Scale bar = 1 cm.

The shape of the mandibular gland of the white‐eared opossum was elliptical‐triangular. It was located ventral to the parotid gland, caudoventral to the masseter muscle and mandibular lymph nodes, and lateral to the sternohyoid muscles (Figure 1).

**Figure 2:**
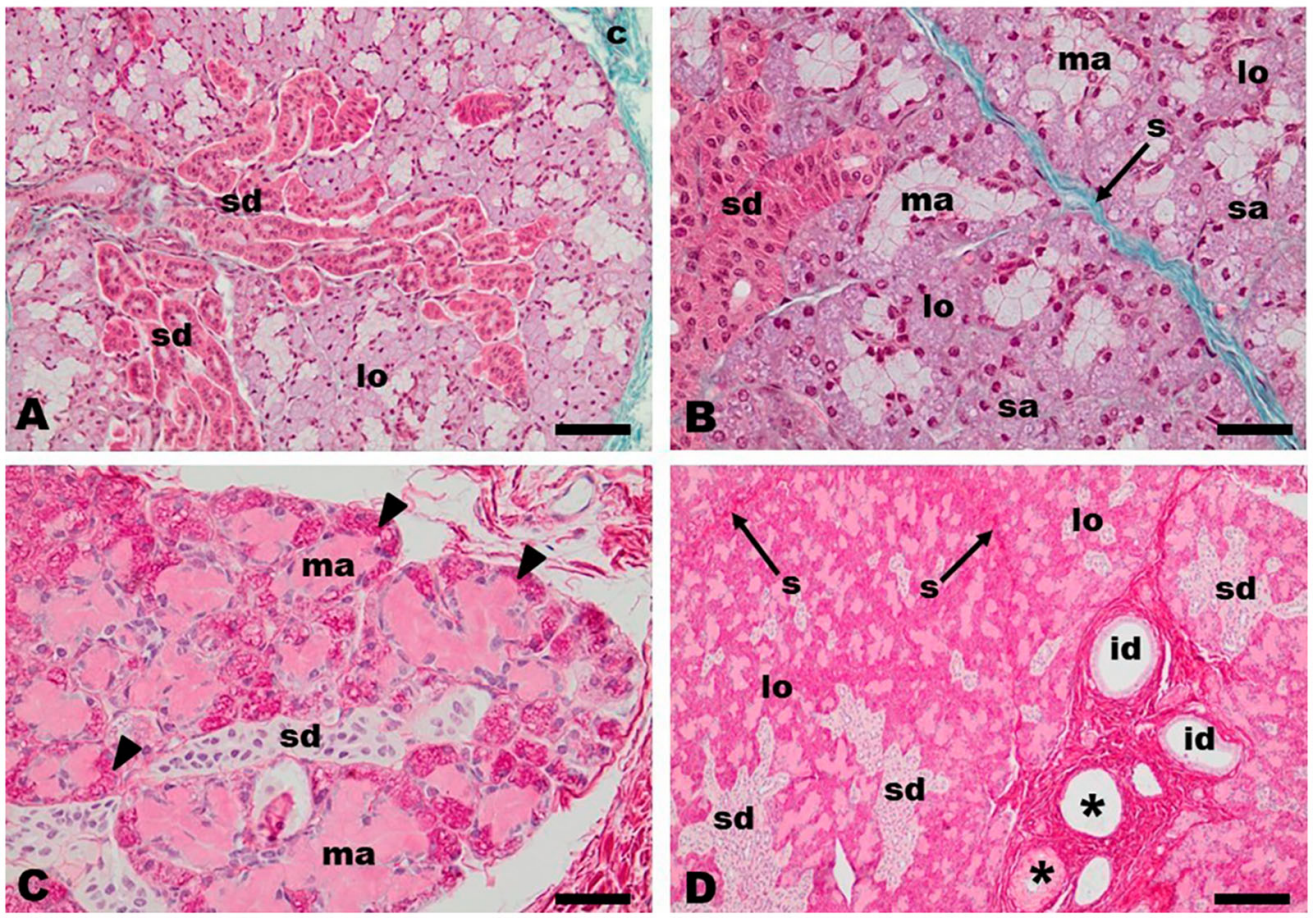
*Didelphis albiventris*, mandibular salivary gland. The connective tissue capsule (c) gives off septa (s) that divide the mandibular gland into lobules (lo). Mucous (ma) and serous acini (sa) constituted the lobules. Note the serous demilune (arrowheads) surrounding mucous acini (ma). Note also the abundance of the striated ducts (sd) and interlobular ducts (id) that drain glandular secretion and the presence of blood vessels (asterisks) in the interlobular connective tissue. Masson's Trichrome (A,B); Haematoxylin and eosin staining (C, D). Scale bar: A = 50 µm, B and C = 20 µm, D = 100 µm.

**Figure 3:**
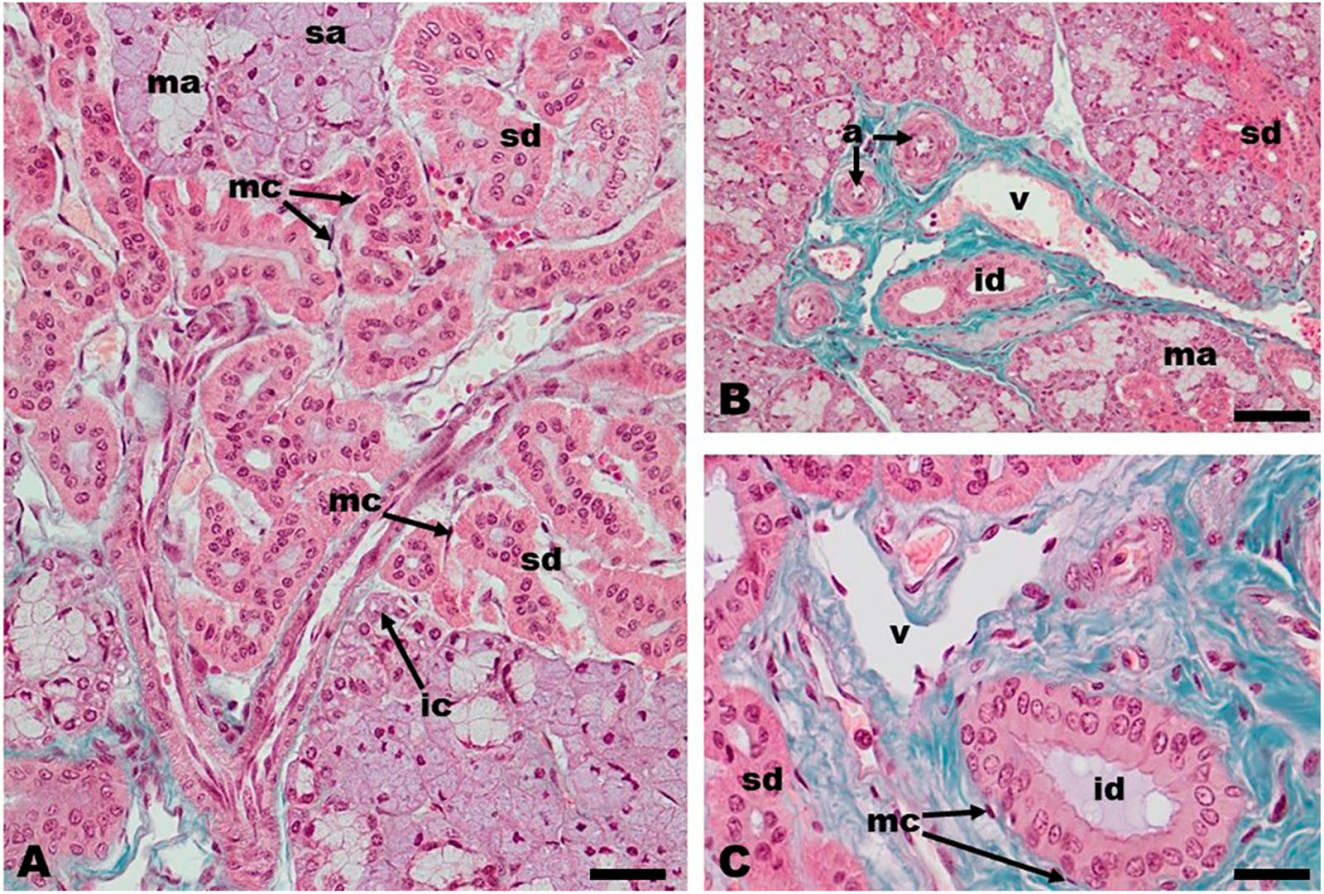
*Didelphis albiventris*, mandibular salivary gland. Note the intercalated ducts (ic), striated ducts (sd), and interlobular ducts (id). Serous (sa) and mucous (ma) acini, and myoepithelial cells (mc) are showed. The blood vessels such as arteries (a) and veins (v) are also observed in the interlobular connective tissue. Masson's Trichrome staining. Scale bar: A and C = 20 µm, B = 50 µm.

The mandibular salivary gland of the white‐eared opossum was enclosed in a connective tissue capsule (Figure 2A) that delimited the glandular parenchyma. This capsule formed septa that reached into the gland, dividing it into lobules (Figure 2B,D). The parenchyma of the glandular lobules consisted of secretory units, the acini, and a duct system (Figures 2A–D; 3A–C). The mandibular salivary gland of the white‐eared opossum was a mixed gland of serous and mucous acini with predominance of serous acini (Figures 2A,B; 3A). The presence of serous and mucous acini appeared somewhat random in the glandular lobules. The mucous acini were partially covered by serous structures in the crescent‐shape arrangement called serous demilune (Figure 2C). Serous acini were made up of cells with mainly rounded nuclei located in the basal part of the acinar cells (Figures 2A,B; 3A,B), even when they formed crescents (Figure 2C). The mucous acini, in turn, presented elongated or avoided nuclei also located in the basal part (Figures 2B,C; 3A).

The duct system that drains the mandibular gland was formed by intercalated, striated, and interlobular ducts (Figures 2A,B,D; 3A–C). These ducts were intraglandular and found in the connective tissue of the glandular stroma. The intercalated and striated ducts were intralobular ducts. The intercalated ducts were the smallest ducts and were lined by simple cubic epithelium (Figure 3A). The striated ducts were so extensive that they constituted the most striking features observed in the mandibular gland of the white‐eared opossum (Figures 2A,B; 3A). The ducts formed true “ductal regions” within the glandular lobule. The striated ducts displayed a larger diameter than the intercalated ducts and were lined with a simple cubic epithelium. Conspicuous nucleoli were observed within their nuclei (Figure 3A). Groups of striated ducts came together to form the interlobular ducts (Figures 2C; 3C). The interlobular ducts were found close to the connective tissue of the interlobular septa of the gland and were always associated with blood vessels such as arteries and veins (Figures 2D; 3B,C). The epithelium lining of the interlobular ducts was a simple columnar epithelium with ovoid nuclei located in the middle part of the cell cytoplasm (Figure 3C). Myoepithelial cells were observed around the epithelium of the striated and interlobular ducts (Figure 3A,C).

### 3.2 Histochemistry of the Mandibular Glands

The secretory acini, striated and interlobular ducts found in the white‐eared opossum exhibited a unique histochemical and lectin profile. None of them reacted to HID. The results of histochemical staining are summarized in Table 2.

**Table 2:** Glycohistochemical pattern of the mandibular gland parenchyma of the white‐eared opossum (*Didelphis albiventris*).

Reactions	Secretory acini	Striated ducts	Interlobular ducts
PAS	+++/+++lc	±lc	±lc
AB pH 2.5	++/+++lc	±lc	±lc
HID	—	—	—
Con A	±	+++as	+ac
RCA_120_	—	—	±lc
PNA	—	++as	+ac
DBA	—	++	++/±lc
SBA	—	—	+/±lc
AAL	+++	+++as	+ac
MAL II	++lc	+	—
SNA	±	+	+ac

*Note:* If not specified, the staining is located in the cytoplasm. Staining intensity: ‐ negative; ±,+,++,+++, faint, weak, moderate, and strong, respectively.

Abbreviations: ac, apical cytoplasm; as, apical surface; lc, luminal content.

**Figure 4:**
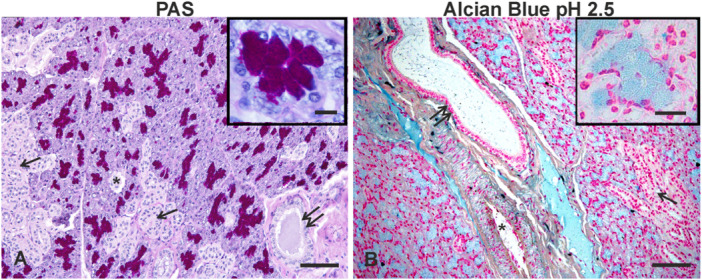
*Didelphis albiventris*, PAS (A) and Alcian blue pH 2.5 (AB 2.5) (B) staining of the mandibular gland. (A) Note the strong PAS‐positivity (magenta) of the secretory acini and the faint staining of intraluminal content of the striated (arrows) and interlobular ducts (double arrows) whose epithelium was PAS negative. The inset shows a detail of the PAS positive reaction in the secretory acini. (B) AB 2.5 staining (azure) was observed in the secretory epithelium and in a minor extent in the lumen of the duct. The inset shows a detail of the AB positive reaction in the secretory acini. ct, connective tissue that surrounds the interlobular duct. The nuclei were stained with Hematoxylin in A and with fast red in B. Asterisk, blood vessel. Scale bar: A = 100 µM; B = 80 µM; inset of A,B = 20 µM.

The PAS staining strongly stained the secretory acini and faintly stained the lumen content of the ductus system (Figure 4A). Except for the weaker staining of the secretory acini, AB 2.5 (Figure 4B) showed a similar staining pattern of PAS (Figure 4B). High‐Iron Diaminedid not stain any glandular structure.

**Figure 5:**
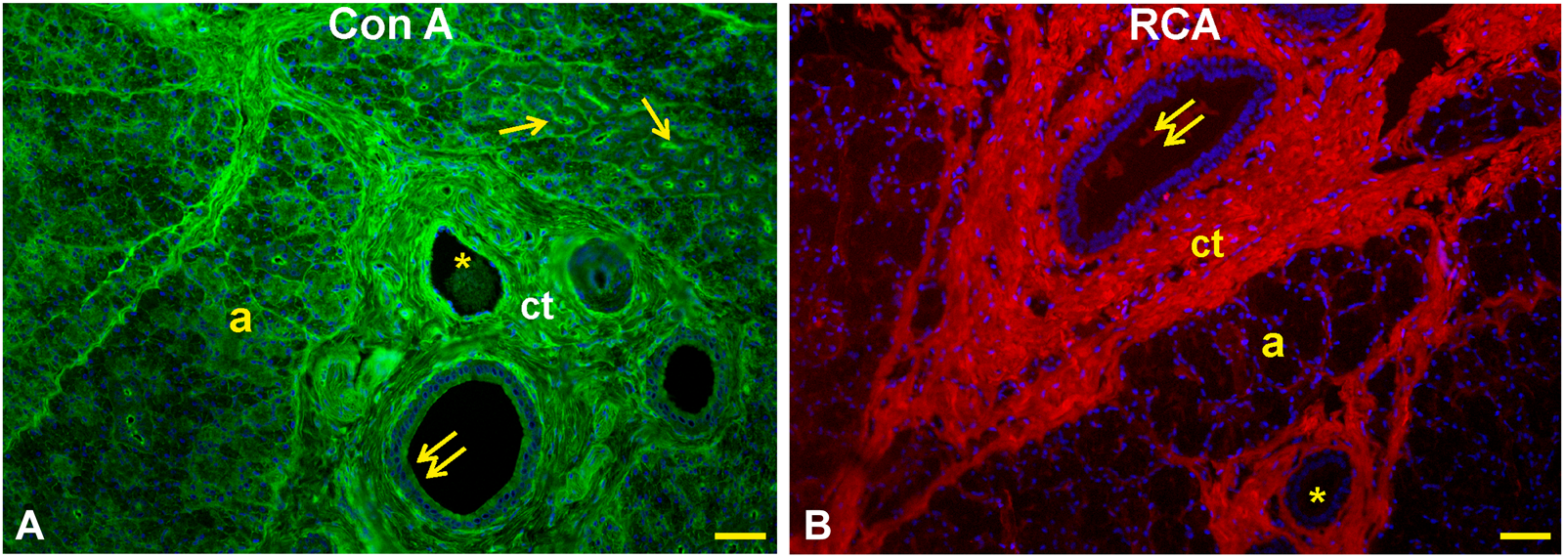
*Didelphis albiventris*, reactivity pattern of Con A (A) and RCA_120_ (B) with the mandibular gland. (A) Con A binding sites are present in the secretory acini (a), apical surface of the epithelium lining of the striated ducts (arrows), and in the epithelium of the interlobular ducts (double arrow). (B) RCA reactivity was observed in the lumen content of the interlobular ducts (double arrows) and in the connective tissue. ct, connective tissue (stroma); asterisks, blood vessels. Scale bar: A, B = 50 µm.

**Figure 6:**
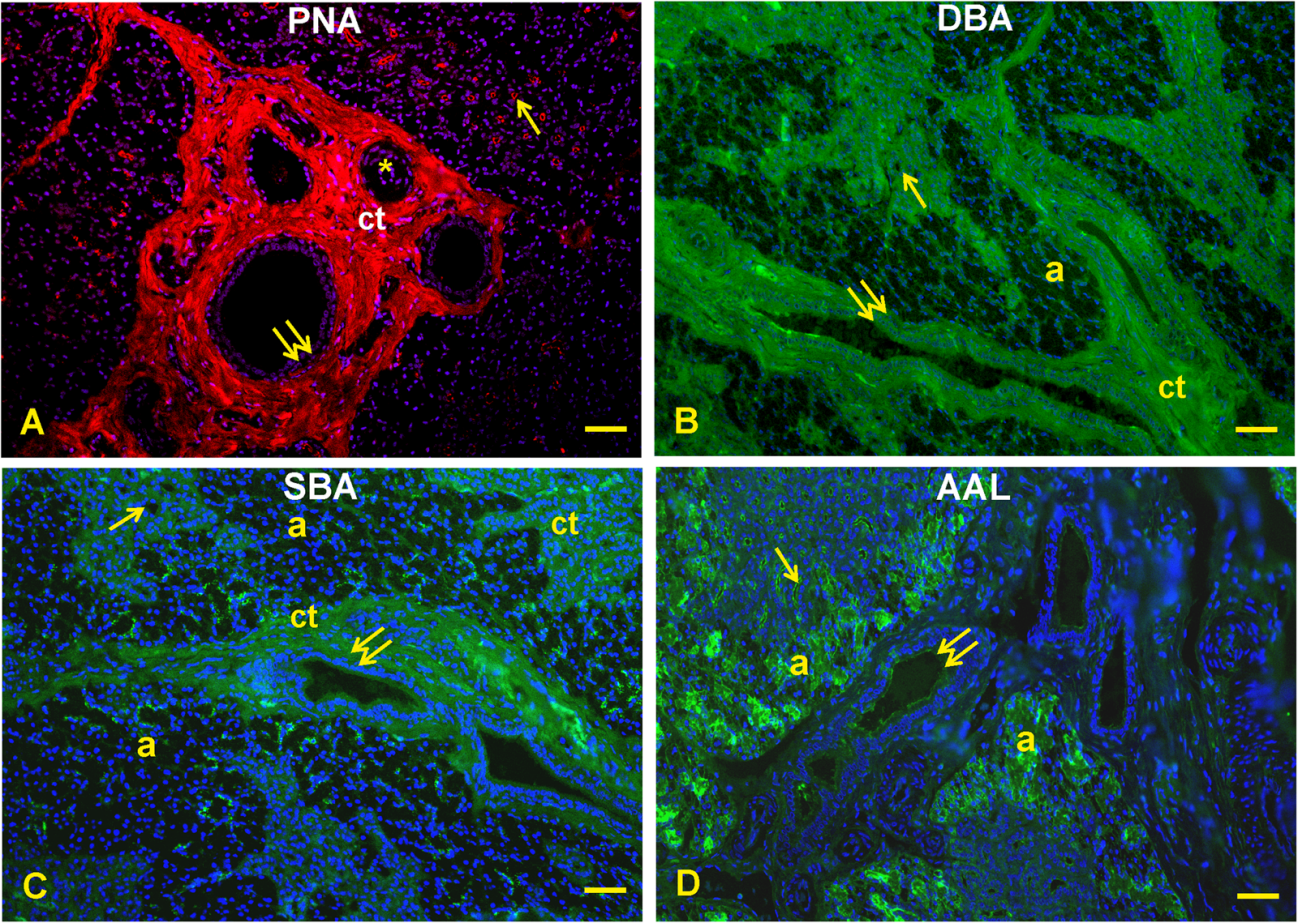
*Didelphis albiventris*, binding pattern of PNA (A), DBA (B), SBA (C), and AAL (D) in the mandibular gland. (A) PNA affinity is observed in the apical surface of the striated ducts (arrows), in the apical cytoplasm of interlobular ducts and in the interlobular connective tissue. (B) Moderate DBA reaction in the epithelium of the striated (arrows) and interlobular (double arrows) ducts. The latter ducts contained a faintly visible positive substance. (C) SBA binding sites are visible in the epithelium of the interlobular ducts (double arrows), which contain a barely reactive substance and are surrounded by a well visible stroma (ct). (D) AAL strongly reacts with the secretory acini (a) and the apical surface of the striated ducts (arrows). A barely AAL reaction is visible in the interlobular ducts (double ducts). ct, connective tissue; asterisks, blood vessels. Scale bar: A, B, C, D = 50 µm.

**Figure 7:**
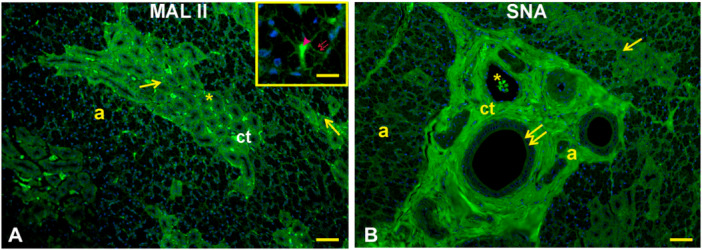
*Didelphis albiventris*, MAL II (A) and SNA (B) binding pattern in the mandibular gland. (A) MAL II reactivity is present in the lumen (arrowhead) and the lateral surface (red double arrows) of the secretory acini (a) and in the striated ducts (arrows). The inset shows a detail of the positive reaction of the acini. (B) SNA binding sites are faintly observed in the secretory acini (a), and weakly in the striated (arrows) and apical cytoplasm of the interlobular (double arrows) ducts. Note the strong staining of the stroma. ct, connective tissue; asterisks, blood vessels. Scale bar: A, B = 50 µm; inset of A = 15 µm.

Lectin histochemistry: Con A (Figure 5A) gave a weak reaction with the secretory cells and the apical cytoplasm of the interlobular duct epithelium. This lectin displayed a strong reactivity in the apical surface of the striated ducts and the connective tissue (stroma) that supports the glandular parenchyma. RCA_120_ (Figure 5B) did not react with the parenchyma of the gland, whereas it stained faintly the lumen content of interlobular ducts and the connective tissue. PNA (Figure 6A) bound the apical surface of the striated ducti and the apical cytoplasm of the interlobular ducts which were surrounded by positively stained connective tissue. DBA (Figure 6B) displayed moderate staining of the epithelium lining of the ducts and the stroma; the interlobular duct contained a substance that was weakly reactive to DBA. SBA (Figure 6C) found few reactive sites in the epithelium and lumen content of interlobular duct, which were enclosed in a strongly positive connective tissue. AAL (Figure 6D) reacted strongly with the secretory acini and the apical surface of the striated ducts and weakly with the supranuclear cytoplasm of the interlobular ducts. MAL II (Figure 7A) moderately reacted with the lumen content of the glandular acini and weakly with the lining epithelium of the striated ducts. MAL binding sites were found in the lateral surface of the acini cells. Lastly, SNA (Figure 7B) reacted faintly with the cytoplasm of cells of the secretory acini and weakly with the epithelium of the striated ducts and the apical cytoplasm of interlobular ducts. The glandular parenchyma was surrounded by a strongly SNA‐positive stroma.

## 4 Discussion

White‐eared opossums (*Didelphis albiventris*) are omnivorous marsupials, with a diet that includes fruits, insects, eggs and small vertebrates (Albert et al. 2020; Bitencourt and Bezerra 2022; Delciellos et al. 2016). Furthermore, they are important seed dispersers, playing a crucial role in urban forest fragments (Cáceres 2002; Cáceres and Monteiro‐Filho 2007; Oliveira and Leme 2013). Saliva secreted by the salivary glands can play an important role in the digestion of fruit seeds (Santos et al. 2013). The salivary glycoproteins are involved in the function of this biological fluid. The present study provides details regarding the gross and microscopic anatomy and the glycan pattern of the white‐eared opossum mandibular gland.

### 4.1 Topography and Histomorphology

The topographic relationships of the mandibular salivary gland in the white‐eared opossum, for example, with the mandibular lymph nodes and the sternohyoid, masseter and digastric muscles, are in agreement with those reported in previous studies with other mammals such as the crab‐eating raccoon (Pereira et al. 2013; Santos et al. 2013), bushy‐tailed opossum (Vieira et al. 2015), red‐rumped agouti (Oliveira Júnior et al. 2016), Spix's yellow‐toothed cavy (Rebouças et al. 2024), and giant anteater (Schimming et al. 2025).

The histoarchitecture of the mandibular gland of *Didelphis albiventris* consists of an outer capsule of dense connective tissue that forms septa into the gland which divide the glandular stroma into lobules. This is a common feature of the mandibular gland in marsupials, such as the bushy‐tailed opossum (*Glironia venusta*) (Vieira et al. 2015) and the koala (*Phascolarctos cinereus*) (Krause 2010), as well as in wild eutherians such as giant‐anteater (*Myrmecophaga tridactyla*) (Schimming et al. 2025), aardvark (*Orycteropus afer*) and lowland tapir (*Tapirus terrestris*) (Klećkowska‐Nawrot et al. 2023), porcupine (*Hystrix indica*) (Estecondo et al. 2005), southern white‐breasted hedgehog (*Erinaceus concolor*) (Massoud et al. 2023), Eurasian wolf (*Canis lupus lupus*) (Klećkowska‐Nawrot et al. 2025), capybara (*Hydrochoerus hydrochaeris*) (Donoso et al. 2024), spix's yellow‐toothed cavys (*Galea spixii* Wagler, 1831) (Rebouças et al 2024), and porcupine (*Hystrix indica*) (Goodarzi and Kahrizi 2023).

The secretory units of the white‐eared opossum mandibular gland are of acinar‐type. The comparison with the secretory parenchyma of the mandibular gland of other marsupials (Table 3) shows that acinar secretory units are present in the omnivorous bushy‐tailed opossum (Vieira et al. 2015) but not in the omnivorous North American opossum (*Didelphis virginiana*) (Wilborn and Shackelford 1969) and in the herbivorous Australian brush‐tail possum (*Trichosurus vulpecula*) (Blood et al. 1977) and koala (*Phascolarctos cinereus*) (Krause 2010), whose secretory parenchyma has a tubule‐acinar appearance. These findings suggest that the morphology of the secretory parenchyma is species‐specific in omnivorous opossums and is similar in herbivorous marsupials such as possums and koalas. No relationship between feeding habits and the morphology of the mandibular gland secretory units has also been reported in wild eutherians (Table 3). For example, the myrmecophagous giant anteater (*Myrmecophaga tridactyla*) (Schimming et al. 2025) and aardvark (*Orycteropus afer*) (Klećkowska‐Nawrot et al. 2023) have acinous and branched tubule‐alveolar secretory parenchyma, respectively. In addition, acinar units constitute the secretory parenchyma of the mandibular gland of the insectivorous armadillo (*Zaedyus pichiy*) (Estecondo et al. (2005), omnivorous hedgehog (*Erinaceus concolor*) (Massoud et al. 2023), herbivourous capybara (*Hydrochoerus hydrochaeris*) (Donoso et al. 2024), spix's yellow‐toothed cavies (*Galea spixii*) (Rebouças et al. 2024), porcupine (*Hystrix indica*) (Goodarzi and Kahrizi 2023), and barking deer (*Muntiacus muntjak*) (Adnyane et al. 2010). Lastly, the secretory parenchyma has a tubular appearance in the carnivorous Eurasian wolf (*Canis lupus lupus*) (Klećkowska‐Nawrot et al. 2025) and the tapir (*Tapirus terrestris*) (Klećkowska‐Nawrot et al. 2023).

**Table 3:** Feeding habits, secretory unit morphology, and secretion type in the mandibular glands of marsupial and wild eutherian animals.

Marsupials	Feesding habits	Secretory units	Secretion type	Authors
**Opossums**				
white‐eared opossum (*Didelphis albiventris*)	Omnivorous	Acini	Mucoserous (↑serous)	This study
North American opossum (*Didelphis virginiana*)	Omnivorous	Branched tubules	Mucoserous (↑mucous)	Wilborn and Shackleford (1969)
bushy‐tailed opossum (*Glironia venusta*)	Omnivorous‐insectivore	Acini	Mucoserous (↑mucous)	Vieira et al. (2015)
**Possums**				
Australian brush‐tail possum (*Trichosurus vulpecula*)	Herbivorous	Tubule‐acinar	Serous	Blood et al. (1977)
**Koala**				
koala(*Phascolarctos cinereus*)	Herbivorous	Tubule‐acinar	Serous	Krause (2010)

Eutherians	Feesding habits	Secretory units	Secretion type	Authors
Giant anteater (*Myrmecophaga tridactyla*)	Myrmecophagous	Acini	Mucoserous (↑mucous)	Schimming et al. (2025)
aardvark (*Orycteropus afer*)	Myrmecophagous	Branched tubulo‐alveolar	Mucoserous	Klećkowska‐Nawrot et al. (2023)
armadillo (*Zaedyus pichiy*)	Insectivore	Acini	Mucoserous (↑mucous acini)	Estecondo et al. (2005)
Hedgehog (*Erinaceus concolor*)	Omnivorousacini	Acini	Mucoserous	Massoud et al. (2023)
Eurasian wolf (*Canis lupus lupus*)	Carnivorous	Branched tubulo‐alveolar	Mucoserous	Klećkowska‐Nawrot et al. (2025)
Capybara (*Hydrochoerus hydrochaeris*)	Herbivorous	Acini	Serous	Donoso et al. (2024)
Spix's yellow‐toothed cavies (*Galea spixii)*	Herbivorous	Acini	Mucoserous	Rebouças et al. (2024)
Porcupine (*Hystrix indica*)	Herbivorous	Acini	Mucous	Goodarzi and Kahrizi (2023)
Tapir (*Tapirus terrestris*)	Herbivorous	Branched tubular	Mucous	Klećkowska‐Nawrot et al. (2023)
barking deer (*Muntiacus muntjak*)	Herbivorous	Acini	Mucoserous (↑mucous acini)	Adnyane et al. (2010)

In addition to the secretory units, the lobes of the mandibular gland have a system of ducts whose function is to eliminate salivary secretion produced by secretory units. This duct system is formed by intercalated, striated, and interlobular (excretory) ducts. The duct system pattern observed in the white‐eared opossum is similar to those reported for the North American opossum (Wilborn and Shackleford 1969) and the Australian brush‐tail possum (*Trichosurus vulpecula*) (Blood et al. 1977) and in eutherian mammals such as the lowland tapir and aardvark (Klećkowska‐Nawrot et al. 2023), white‐breasted hedgehog (Massoud et al. 2023), spix's yellow‐toothed cavy (Rebouças et al. 2024), and giant anteater (Schimming et al. 2025). A feature that deserves to be highlighted is the abundance of striated ducts inside the lobules of the mandibular gland of the white‐eared opossum. These ducts were many and formed “ductal regions” inside the glandular lobules. This finding is consistent with those reported for the North American opossum (Wilborn and Shackleford 1969) and Australian brush‐tail possum (*Trichosurus vulpecula*) (Blood et al. 1977). Junqueira (1967) reported that striated ducts are more developed in monotremes and marsupials when compared to most eutherian mammals.

After being elaborated by the secretory units, whether serous, mucous and/or seromucosal acini, saliva passes through the duct system and is modified by the epithelial cells of these ducts (Tandler et al. 2001). The striated ducts are the most prominent of the intralobular ducts. These ducts probably perform physiological functions such as secretion that contributes to water and electrolyte balance and control of saliva pH (Tandler et al. 2001). Moreover, the ductal cells alter salivary composition; for example, cells of the striated ducts use active and passive transport of ions to remove sodium and chloride ions from and add bicarbonate and potassium ions to the primary saliva (Gartner 2021).

### 4.2 Histochemistry

The present study demonstrated that the secretory acini of the mandibular gland of the white‐eared opossum (*Didelphis albiventris*) stained with PAS and AB procedures but not with HID, indicating the production of neutral and acidic non‐sulfated glycans. The copresence of both neutral and acidic mucosubstances is not a common feature in the mandibular gland of the opossums, because acidic mucosubstances are absent in the mucoserous secretory units of the North American opossum *(Didelphis virginiana*) (Pinkstaff 1975). These findings suggest that the type of mucosubstance secreted by the opossum mandibular glands depends on the diet and the habitat. Also, in other marsupial species, a different composition of glycans synthesized by the secretory parenchyma has been reported. The mandibular gland of the Australian brush‐tailed possum (*Trichosurus vulpecula*) produces both neutral and acidic glycans (Blood et al. 1977), which are absent in the serous cells of the koala (*Phascolarctos cinereus*) (Krause 2010).

The secretion of neutral and acidic mucins has also been reported in the mandibular gland of eutherian species such as the mymercophagous giant anteater (Schimming et al. 2025), and aardvark (Klećkowska‐Nawrot et al. 2023), the insectivorous armadillo (*Zaedyus pichiy*)(Estecondo et al. 2005), the omnivorous white‐breasted hedgehog (Massoud et al. 2023), the carnivorous free‐rangeing Eurasian wolf (*Canis lupus lupus*) (Klećkowska‐Nawrot et al. 2025), the herbivorous lowland tapir (Klećkowska‐Nawrot et al. 2023), the spix's yellow‐toothed cavy (Rebouças et al. 2024), and barking deer (*Muntiacus muntjak*) (Adnyane et al. 2010). All these results suggest that the composition of mucins secreted by the mandibular gland is species‐specific. However, a high content of acid‐sulfated glycans has been reported in the mandibular acini of aardvark and lowland tapir (Klećkowska‐Nawrot et al. 2023) and southern white‐breasted hedgehog (Massoud et al. 2023). These observed differences between white‐eared opossum and lowland tapir, aardvark, and southern white‐breasted hedgehog could depend on the staining method used to evidence the sulfated glycans. In the present study, we used the HID staining to demonstrate carbohydrates containing sulfate esters (Spicer 1965), whereas Klećkowska‐Nawrot et al. (2023) and Massoud et al. (2023) used Hale's dialyzed iron method and aldehyde fuchsin, respectively. The effectiveness of the HID method for demonstrating the presence of sulfated mucins has been well demonstrated in a number of studies (Accogli et al. 2018; D'Alessandro et al. 2025; Desantis et al. 2019; Pedini et al. 2000; Spicer et al. 1965).

The lectin affinity of the secretory acini of the white‐eared opossum mandibular gland revealed that the acinous cells produce high‐mannose N‐linked glycans containing terminal residues of α2,6‐linked sialic acid and fucose (reactivity for Con A, SNA, and AAL) (Bojar et al. 2022). This glycan pattern is typical of the mandibular gland of the white‐eared opossum because it has not been observed in the mandibular gland of other nonhuman mammals such as the fallow deer (Pedini et al. 1997), pig (Pedini et al. 2000), horse (Scocco and Pedini 2006), and giant anteater (Schimming et al. 2025).

The lumen content of secretory acini displayed MAL II binding sites. This suggests that the primary saliva contains O‐linked glycans with terminal α2,3‐sialic acid residues (Bojar et al. 2022). Sialoderivatives impart viscoelastic properties to saliva and are involved in food bolus formation and lubrication, thus aiding food swallowing (Cros and Ruhl 2018). The presence of sialomucous glycoproteins containing the terminal NeuAcα2‐6GalNAc sequence (sialyl‐Tn) has been detected in the fractions of the armadillo mandibular gland (Wu et al. 1995). It has been suggested that the salivary acidic mucus constitutes a sticky substance that helps the armadillo gather the small insects composing its diet (Wu et al. 1979). Furthermore, it has been reported that the salivary sialoglycans are involved in the agglutination of multiple bacteria and the removal of terminal sialic acids abolishes agglutination of some oral bacteria (Kozak et al. 2016; Lamont et al. 2010).

The epithelium lining the striated ducts displayed both N‐linked glycans (Con A, AAL, and SNA reactivity) and O‐linked glycans (PNA, DBA, and MAL II affinity). The binding sites for PNA and DBA reveal the presence of terminal Galβl,3GalNAc (PNA affinity) and GalNAc (DBA positivity) residues that are not expressed in acinar cells. The luminal contents did not react with the lectins used. This finding could depend on the small lumen size of these ducts. The presence of Con A, PNA, DBA, fucosylated and sialylated glycans has been reported in the epithelium of striated ducts of mandibular glands of fallow‐deer (Pedini et al. 1997), whereas PNA, DBA, SNA, and Mal II binding sites were not found in the mandibular glands of pigs (Pedini et al. 2000) and horses (Scocco and Pedini 2006). All sugars analyzed in the present study were found in the striated ducts of the giant anteater mandibular gland (Schimming et al. 2025).

Compared with the striated ducts, the epithelium lining the interlobular ducts also expresses SBA binding sites that identify Forssman β‐GalNAc and α‐GalNAc terminated antigen (Bojar et al. 2022), while lacking α2,3‐linked sialic acid glycans (negative MAL II reaction). The lumen content glycans of the interlobular ducts maintained the faintly visible AB pH 2.5 and PAS positivity and reacted with DBA, SBA and RCA_120_. This finding suggests that the saliva of the interlobular ducts contains acidic glycans as well neutral glycans terminating with GalNAc and N‐acetyllactosamine (RCA_120_ positivity) (Bojar et al. 2022). Interestingly, DBA, SBA and RCA_120_ reactivity was not detected in the secretory acini. This finding indicates that the interlobular ducts could be implicated in the chemical composition of the saliva glycoconjugates of the white‐eared opossum. Unfortunately, it is not possible to compare these results with other studies in nonhuman mammals because in most of those investigations either only PAS and AB pH 2.5 staining was used (Klećkowska‐Nawrot et al. 2023) or the composition of glycans present in the lumen of interlobular ducts was not investigated.

The only study reporting the composition of intraluminal glycans of the interlobar ducts of the mandibular gland using traditional glycohistochemistry and lectin histochemistry was performed on the giant anteater (Schimming et al. 2025). Comparison between the results of the present study and that study reveals a different composition of intraluminal glycans because binding sites for Con A, PNA, AAL, MAL II, and SNA binding sites were detected in the interlobar ducts lumen of giant anteater mandibular gland (Schimming et al. 2025)

In conclusion, this study demonstrates that the mandibular gland of the white‐eared opossum (*Didelphis albiventris*) has histological and molecular characteristics that distinguish it from other marsupial and eutherian mammals. Furthermore, for the first time, the contribution of the duct system to the composition of salivary glycoproteins has been demonstrated in marsupials. The observed glycosylation pattern is probably related to the specific diet of the white‐eared opossum which encompasses generally invertebrates, fruits and vertebrates. These results provide new data on the mandibular gland and suggest that all the structures of the glandular parenchyma play a role in the final molecular composition of saliva. Therefore, the results, in addition to providing new data on the mandibular gland of the white‐eared opossum (*Didelphis albiventris*), suggest that all structures of the glandular parenchyma (secretory units and ducts) play a role in the final molecular composition of saliva and stimulate further studies to examine and understand the salivary glands from a more comprehensive perspective.

## Author Contributions

**Bruno Cesar Schimming:** conceptualization, investigation, writing – original draft, writing – review and editing. **Aline Herrera Farha:** investigation and methodology. **Tais Harumi de Castro Sasahara:** investigation, methodology, and validation. **Fabio Cesar Magioli Abdala:** investigation, methodology. **Attilio Cianciotta:** methodology. **Silvio Pires Gomes:** investigation and methodology. **Salvatore Desantis:** conceptualization, investigation, supervision, writing – original draft, writing – review and editing.

## Conflicts of Interest

The authors declare no conflicts of interest.

## Data Availability

The data that support the findings of this study are available on request from the corresponding author.
